# Long-lasting activity of trabectedin in refractory uterine leiomyosarcoma: a case report

**DOI:** 10.1186/s12885-015-2038-7

**Published:** 2015-12-22

**Authors:** Alberto Bongiovanni, Nada Riva, Marianna Ricci, Laura Mercatali, Chiara Liverani, Federico La Manna, Alessandro De Vita, Davide Cavaliere, Federica Pieri, Devil Oboldi, Giorgio Maria Verdecchia, Dino Amadori, Toni Ibrahim

**Affiliations:** 1 Osteoncology and Rare Tumors Center, Istituto Scientifico Romagnolo per lo Studio e la Cura dei Tumori (IRST) IRCCS, Meldola, Italy; 2 Unit of Surgery and Advanced Oncologic Therapies, Morgagni-Pierantoni Hospital, Forlì, Italy; 3 Pathology Unit, Morgagni-Pierantoni Hospital, Forlì, Italy; 4 Radiology Unit, IRST IRCCS, Meldola, Italy

**Keywords:** Uterine leiomyosarcoma, Soft tissue sarcoma, Trabectedin

## Abstract

**Background:**

Leiomyosarcoma (LMS) is an aggressive soft tissue sarcoma derived from smooth muscle cells typically of uterine, gastrointestinal or soft tissue origin.

The prognosis for this tumor is poor, with survival rates among the lowest of all soft tissue sarcomas. Surgery is the best approach for localized disease. The principal role of chemotherapy is prevalently in the treatment of metastatic disease. Trabectedin, a promising new DNA-damaging agent with a mechanism of action that differs from that of traditional alkylating agents, has been approved in Europe for the treatment of patients with advanced soft tissue sarcoma after failure of anthracyclines and ifosfamide,

**Case presentation:**

We report the case of a 53-year-old woman with metastatic well differentiated uterine leiomyosarcoma refractory to multiple treatments who underwent 22 cycles of trabectedin over 30 months, obtaining a partial response according to RECIST (Response Evaluation Criteria in Solid Tumors) criteria, with good tolerability, and maintaining the response for 10 months after trebectedin withdrawal.

**Conclusion:**

This very prolonged response, which persisted after drug discontinuation, suggests that trabectedin exerts an oncostatic effect rather than the cytotoxic one produced by other chemotherapeutic agents. Our experience also raises the question of the best way to evaluate trabectedin efficacy.

## Background

Leiomyosarcomas (LMSs) constitute a wide group of histopathologically heterogeneous tumors derived from smooth muscle cells. These aggressive malignancies represent one of the most common types of soft tissue sarcoma, with an incidence of 11 % [1]. LMSs are of uterine origin in 50 % of cases but have also been reported in the kidneys [2], pancreas [3], retroperitoneum [4], thyroid [5], lungs [6], blood vessels [7] and skin [8]. In addition to various sites of origin, LMSs also show substantial variability from a histological and morphological point of view and are classified into the following subtypes: myxoid [9], epithelioid [10], dedifferentiated [11], inflammatory [12], granular cell [13] and well differentiated [14]. In the majority of cases, diagnosis is made following hysterectomy or myomectomy performed for presumably benign leiomyoma [15]. A high incidence of local and distant relapse has also been reported, even after complete resection [16].

The factors associated with poor prognosis include age >62 years, tumor size > 4 cm, tumor necrosis, Fédération Nationale des Centres de Lutte Contre le Cancer (FNCLCC) grade, vascular invasion, or previous intralesional surgery [17, 18]. Although the mitotic index is not directly correlated with a worse outcome, it is clearly a useful parameter to distinguish between malignant and benign tumors. Furthermore, in a retrospective study of 66 patients with LMS, Mankin et al. reported that Musculoskeletal Tumor Society (MSTS) stage and size had a significant impact on outcome, whereas gender, age, site, adjuvant therapy and presence of local recurrence did not. Overall survival (OS) reported for patients diagnosed with LMS ranges from 50 % at 3 years to 64 % at 5 years, making this tumor one of the most aggressive forms of soft tissue sarcoma (STS) [19].

The primary role of chemotherapy in LMS is to treat metastatic disease; although not curative, it has been shown to slow down disease progression. Drugs frequently used include doxorubicin and ifosfamide, gemcitabine and taxotere (docetaxel), dacarbazine, and trabectedin. Some reviews have reported mortality rates ranging from 77 % to 93 % for retroperitoneal/abdominal leiomyosarcoma [20]. However, a number of studies with small case series have provided a valuable insight into the prognostic significance of some patient variables. A phase III clinical trial comparing adjuvant chemotherapy composed of cisplatin, ifosfamide and doxorubicin followed by radiotherapy with radiotherapy alone in patients with localized uterine sarcomas reported promising results in favor of combined modalities, with a 3-year improvement in progression-free survival (PFS) [21]. Other interesting findings were obtained in a prospective phase II trial investigating the combination of gemcitabine and docetaxel followed by doxorubicin in stage I/II LMS, the authors observing a 2-year PFS of 78 % [22]. In a study by Schmitt et al., patients with unresectable leiomyosarcoma (*n* = 34), mainly of the uterine type, received gemcitabine and docetaxel in combination [23]. Sixteen of the patients were treated after progressing on doxorubicin-based therapy, obtaining a 53 % response rate and a median time to progression (TTP) of 5.6 months. Hensley et al. used the same regimen as second-line treatment for patients with advanced uterine LMS [24]. Although the objective response rate (ORR) (27 %) was lower than that of the first trial, an additional 50 % of patients had stable disease (SD) for a median of 5.4 months and the 24-week PFS rate was 52 %. Options in advanced or relapsed stages were evaluated in a phase II clinical trial investigating ridaforolimus (mTOR inhibitor) in bone and soft tissue sarcomas, and a subsequent phase III trial analyzed the efficacy of the same drug as maintenance therapy [25]. Furthermore, a non-randomized phase II clinical trial evaluating eribulin in patients with advanced or metastatic sarcomas reported the best treatment response to date [26]. Finally, results from a randomized placebo-controlled phase III trial in patients with metastatic and recurrent soft tissue sarcomas treated with pazopanib revealed an increase in PFS with respect to placebo [27].

Trabectedin (Yondelis®; PharmaMar, Madrid, Spain), is a cytotoxic agent originally isolated from the Caribbean sea squirt *ecteinascidia turbinata* and is currently manufactured by total synthesis. With pleiotropic mechanisms of action, it binds to the DNA minor groove, causing double helix bending towards the major groove, modulating inflammatory responses in the tumor microenvironment, and promoting tumor cell differentiation [28]. The pivotal registration study of trabectedin was a randomized phase II comparison (ET-743-STS-201 trial) of two different schedules of trabectedin administered either every 3 weeks (q3w) or weekly in anthracycline- and ifosfamide-pretreated patients with LMS or liposarcoma [29]. Data from the trial showed that trabectedin 1.5 mg/m^2^ given as a 24-h intravenous infusion in a q3w-regimen obtained better disease control than weekly trabectedin 0.58 mg/m^2^ (3-h infusion for three consecutive weeks in a 4-week cycle) in terms of longer TTP (median TTP: 3.7 vs. 2.3 months; *P* = 0.0302) and PFS (median PFS: 3.3 vs. 2.3 months; *P* = 0.0418). Based on these results, in 2007 trabectedin was approved for the treatment of patients with advanced STS following failure of anthracyclines and ifosfamide, and also for those who were not amenable to treatment with these drugs. A recent phase III trial showed no significant difference in PFS or OS between trabectedin and doxorubicin-based chemotherapy in selected patient populations [30]. The histological heterogeneity of STS, compounded by their rarity, poses particular challenges in personalizing therapy.

In the absence of large randomized studies, institutional case series can provide useful insights into the efficacy, toxicity and management of patients receiving novel agents. In this paper we describe the case of a 53-year-old woman with LMS in progression after several treatments who obtained a durable response with trabectedin that persisted after the drug was withdrawn.

## Case presentation

**Fig. 1:**
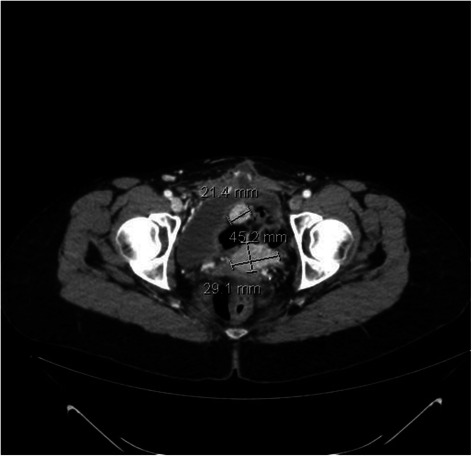
January 2011: CT scan showing peritoneal progression after gemcitabine

We report the case of a 53-year-old woman who was referred to our Institute with a history of myomectomy in 1997, 2003 and 2004, and hysterectomy in 2006 following a diagnosis of well differentiated, estrogen receptor (ER)-positive uterine LMS. In November 2006 a CT scan revealed several peritoneal lesions which were surgically removed. The pathology report described several localizations of well differentiated uterine leiomyosarcoma (20 % estrogen and 80 % progesterone receptor-positive). Five cycles of chemotherapy with adriamycin and ifosfamide were administered, with no signs of recurrence. In 2007, the patient underwent ovariectomy and metastasectomy for multiple lung lesions and then entered follow-up. In 2009, a CT scan showed abdominal and rectal recurrence and the patient was submitted to omentectomy with the removal of omental nodules, mesosigma and rectum. Somatostatin-receptor scintigraphy (Octreoscan) revealed positivity of abdominal lesions and the patient was enrolled onto a phase I clinical trial, receiving 5 cycles of 177Lu radioreceptor DOTATATE. Treatment lasted from September 2009 to May 2010, with a cumulative dose of 610 mCi. CT evaluation after the last treatment cycle showed disease progression in the pleura, lungs and abdomen. It was thus decided to start weekly gemcitabine alternating with 3 cycles of peritoneal hyperthermia, the latter performed in a different institution. After two cycles of chemotherapy, a CT scan showed peritoneal progression (Fig. 1) and stability of the lung metastases. In March 2011 the patient began treatment with trabectedin at a dose of 1.5 mg/m^2^ administered as a 24-h intravenous infusion every 3 weeks. Each infusion was preceded by 500 ml of 0.9 % sodium chloride and 20 mg dexamethasone given intravenously and followed by a short course of prednisone.

**Fig. 2:**
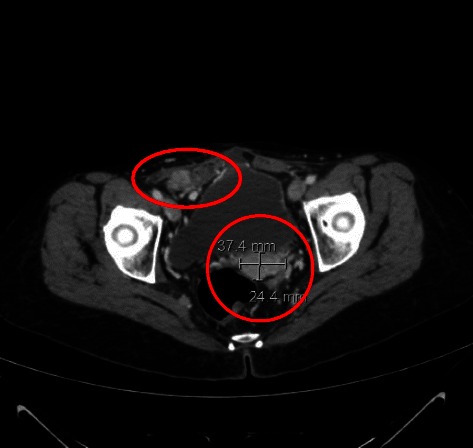
July 2012 (11 months after the start of treatment): CT scan showing a reduction in the size of the abdominal lesions

**Fig. 3:**
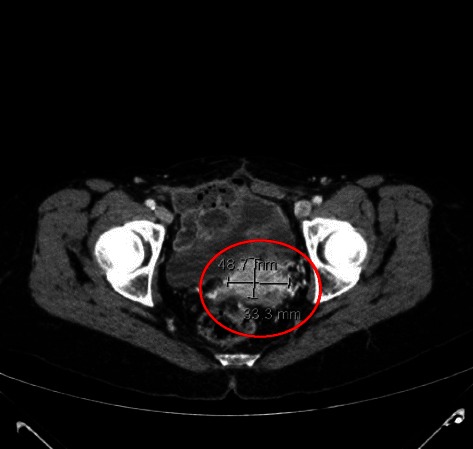
September 2013: CT scan showing abdominal progression with an increase in the size of the peritoneal lesions

Treatment with trabectedin continued until November 2012 (total of 22 cycles), the patient obtaining a partial response according to RECIST criteria confirmed 11 months after the start of treatment (Fig. 2). Hematological toxicity was limited to grade 1-2 reversible neutropenia and an isolated episode of grade 3 hyper-transaminasemia after the third cycle of treatment. In October 2012, after a CT scan revealed a slight dimensional increase in the abdominal lesions, the patient decided to ask for a second opinion at another institute where it was proposed to withdraw trabectedin and begin a new course of chemotherapy with dacarbazine for 3 cycles. In December 2012, before starting dacarbazine, the patient underwent a CT scan which confirmed the stability of the peritoneal disease but highlighted an increase in the size of a previously documented single lesion in the right abdominal wall. Surgery was performed and a histological diagnosis was made of grade 3 leiomyosarcoma according to FNCLCC criteria with several necrotic foci representing less than 50 % of the lesion. Two months later, imaging studies re-confirmed stable disease in the peritoneum. However, in September 2013, 10 months after the last cycle of trabectedin and 30 months after the start of treatment, a CT scan showed abdominal and peritoneal progression (Fig. 3), and it was decided to proceed with pazopanib. Treatment is ongoing.

## Discussion

Leiomyosarcoma is an aggressive STS derived from smooth muscle cells typically of uterine or gastrointestinal origin. STS have a number of histologic subtypes including epithelioid, myxoid, inflammatory, granular cell and dedifferentiated, but the clinical impact of these subtypes has not been widely investigated. The present report describes the rare case of a patient with heavily pretreated metastatic uterine LMS who received trabectedin and showed a partial response according to RECIST criteria that persisted for 10 months after drug withdrawal (30 months after the start of treatment), with good tolerability.

Trabectedin is a substance derived from a type of marine invertebrate. Its mechanisms of action are based on an interaction with the minor groove of the DNA double helix which affects gene transcription and DNA repair pathways, resulting in G2-M cell cycle arrest and ultimately apoptosis. It is currently used in previously treated advanced soft-tissue sarcoma. The median overall survival of patients with STS is 6 months in unresectable or metastatic disease that progresses after treatment with anthracyclines and ifosfamide. Recently, a retrospective analysis of 66 patients with metastatic uterine LMS, the majority in progression after 2-3 different treatments, reported that 11 patients achieved a radiological partial response according to RECIST criteria and a further 23 demonstrated stable disease following treatment with trabectedin [31]. Galizia et al. described a rare case of 17-month disease stability in a 76-year-old patient with progressive metastatic lung lesions from previously resected primary LMS of the thigh who underwent third-line treatment with 22 cycles of trabectedin [32].

Conventional chemotherapy is usually administered for a limited period because its efficacy decreases over time and because of cumulative toxicities. In particular, cardiotoxicity from anthracycline-based regimens and renal and neuronal toxicities from other chemotherapeutic agents such as ifosfamide and cisplatin can negatively affect subsequent therapeutic options and quality of life [33–35]. In our case, the main toxicities reported were mild neutropenia and reversible hyper-transaminasemia, in agreement with the safety profile of trabectedin. This allowed trabectedin to be administered for a long period without cumulative toxicity and with an acceptable quality of life for the patient. Disease control was maintained over time, impacting overall survival.

The very prolonged response obtained suggests that trabectedin may be capable of keeping tumor cell growth under control and is indicative of an oncostatic effect rather than the cytotoxic action previously associated with this drug. Recent data have shown that trabectedin selectively targets mononuclear phagocytes, including tumor-associated macrophages, and downregulates the production of pro-inflammatory mediators which induces changes in the tumor microenvironment and contributes to its antitumor activity. This immunomodulating effect with high anti-inflammatory and antiangiogenic activity may explain the durable response experienced by our patient.

## Conclusions

The low toxicity profile of trabectedin permitted us to continue treatment for a lengthy period and the response achieved was maintained by the patient for several months after its withdrawal. Thus, decreased tumor volume is clearly not the only criteria with which to define trabectedin activity. Future clinical trials could investigate the methods to define response to trabectedin and it could also be hypothesized to use the drug as maintenance therapy to positively impact the overall survival of patients with soft tissue sarcoma.

## Consent

Written informed consent was obtained from the patient for publication of this case report and any accompanying images. A copy of the written consent is available for review by the Editor of this journal.

## Abbreviations

LMS: Leiomyosarcoma
STS: Soft tissue sarcoma
FNCLCC: Fédération Nationale des Centres de Lutte Contre le Cancer
PFS: Progression-free survival
ORR: Objective response rate
SD: Stable disease
TTP: Time-to-progression
OS: Overall survival
CT: Computerized tomography
MSTS: Musculoskeletal tumor society
RECIST: Response evaluation criteria in solid tumors
